# Low pretreatment prognostic nutritional index predicts poor survival in breast cancer patients: A meta-analysis

**DOI:** 10.1371/journal.pone.0280669

**Published:** 2023-01-20

**Authors:** Guoming Hu, Qiannan Ding, Kefang Zhong, Shimin Wang, Songxiang Wang, Liming Huang

**Affiliations:** 1 Department of General Surgery (Breast and Thyroid Surgery), Shaoxing People’s Hospital, Shaoxing Hospital, Zhejiang University School of Medicine, Shaoxing, Zhejiang, China; 2 Key Laboratory of Cancer Prevention and Intervention, Ministry of Education, Hangzhou, Zhejiang, China; 3 Shaoxing Key Laboratory of Functional Molecular Imaging of Tumor and Interventional Diagnosis and Treatment, Shaoxing, Zhejiang, China; 4 Medical Research Center, Shaoxing People’s Hospital; Shaoxing Hospital, Zhejiang University School of Medicine, Shaoxing, Zhejiang, China; 5 Department of Nephrology, Shaoxing People’s Hospital, Shaoxing Hospital, Zhejiang University School of Medicine, Shaoxing, Zhejiang, China; Shuguang Hospital, CHINA

## Abstract

**Background:**

Prognostic nutritional index (PNI), as an indicator of nutritional immune status, has been shown to be associated with therapeutic effects and survival of solid tumors. However, the prognostic role of PNI before treatment in human breast cancer (BC) is still not conclusive. Hence, we performed this meta-analysis to assess the value of it in prognosis prediction for BC patients.

**Materials and methods:**

We searched PubMed, Embase, Web of Science and EBSCO to identify the studies evaluating the association between PNI and survival such as overall survival (OS), disease–free survival (DFS) of BC, and computed extracted data into hazard ratios (HRs) for OS, DFS and clinicopathological features with STATA 12.0.

**Results:**

A total of 2322 patients with BC from 8 published studies were incorporated into this meta-analysis. We discovered that low pretreatment PNI was significantly associated with worse OS, but not with DFS in BC patients. In stratified analyses, the result showed that decreased PNI before treatment was remarkably related with lower 3-year, 5-year, 8-year and 10-year OS, but not with 1-year survival rate in BC. In addition, although reduced PNI could not impact 1-year, 3-year or 5-year DFS, it considerably deteriorated 8-year and 10-year DFS in patients.

**Conclusion:**

Low pretreatment PNI deteriorated OS, 8-year and 10-year DFS in BC patients, implicating that it is a valuable prognostic index and improving the nutritional immune status may offer a therapeutic strategy for these patients.

## Introduction

Breast cancer (BC) has surpassed lung cancer as the most common malignancy in the world [1]. Although progresses in early diagnosis and therapeutic strategies such as surgery, adjuvant chemotherapy, endocrine, Her-2-targeted and anti-PD1/PD-L1 therapies et al have benefited these patients, the advances in prediction of prognosis remain disappointing. Apart from that age, tumor size, lymph node status, tumor differentiation, molecular type such as triple-negative et al and Ki-67 have been considered as traditional biomarkers which are associated with prognosis of BC, recent study has reported that host status including nutrition or inflammation could be regarded as a prognostic factor of BC [2]. In addition, nutritional immune status has been broadly investigated to risk-stratify cancer patients to improve treatment strategy and to predict clinical outcomes in diverse tumors including BC.

Prognostic nutritional index (PNI), a serum albumin-and peripheral blood lymphocyte-based nutritional parameter, was initially applied to appraise nutritional status in patients undergoing gastrointestinal surgery [3] and predict postoperative complications including intra-abdominal abscess, inflammation of the intestine and obstruction of the intestine. Currently, the PNI has been extended to the field of cancer as an indicator of nutritional immune status and has been shown to be remarkably related with therapeutic effects and clinical outcome of various solid tumors [4–7]. Recently, many researchers have investigated the value of PNI before treatment for BC patients in terms of prognostic prediction [8–10], however, their results were not consistent. A re-assessment is therefore warranted. Moreover, the potential of pretreatment PNI as a valid prognostic index and therapeutic strategy is necessary to be explored.

In this study, we carried out the meta-analysis to quantitatively summarize the association between pretreatment PNI and clinical outcomes such as OS and DFS in BC patients, and thereby provided more evidence on the clinical value of PNI as a prognostic index for BC.

## Methods

### Search strategy

This meta-analysis has been registered in PROSPERO platform (ID: CRD42022349726) *(**https*:*//www*.*crd*.*york*.*ac*.*uk/PROSPERO/**)*. We searched PubMed, Embase, Web of Science and EBSCO for studies assessing the PNI before treatment and survival in BC patients from 1996 to August 31th 2021. The keywords adopted for search were (prognostic nutritional index [All Fields]) AND (breast [Title/Abstract] OR mammary [Title/Abstract]) AND (neoplasms [Title/Abstract] OR tumor [Title/Abstract] OR cancer [Title/Abstract] OR carcinoma [Title/Abstract]). A total of 535, 748, 612 and 1526 entries were identified in PubMed, Embase, Web of Science and EBSCO respectively.

### Inclusion and exclusion criteria

Inclusion criteria of the meta-analysis were: studies must have (1) been published as original articles in English; (2) assessed human subjects with histopathologically diagnosed with BC; (3) provided hazard ratios (HRs) with 95% confidence interval (CI), or Kaplan–Meier curves of high and low PNI before treatment with OS or DFS.

The exclusion criteria were that studies have not been published as research articles including commentary, case report and letters to editors; Studies without sufficient data for hazard ratios (HRs) evaluation; We also excluded studies that detected PNI after treatment such as surgery, chemotherapy, radiotherapy, endocrine and targeted therapy.

### Endpoints

In this meta-analysis, we recorded OS as the primary endpoint; while DFS was regarded as the second endpoint. Individual studies defined cut-offs of PNI and classified BC patients into high- and low- groups.

### Data extraction

Two authors (GM.H. and QN.D.) independently reviewed and extracted information including first author’s name, number of patients, median age, time of follow-up and cut-off value of high PNI. OS, DFS and clinicopathological data such as estrogen receptor (ER), progesterone receptor (PR) and Her-2 status etc were extracted from the text or tables.

### Quality assessment

Two independent authors applied Newcastle–Ottawa Scale (NOS) [11] to appraise the quality of individual study, and achieved consensus for each item under the help of the third or more authors. Six or above that the study scored was considered as high quality.

### Statistical analysis

Relevant data were combined into hazard ratios (HRs) for OS, DFS, and odds ratios (ORs) with STATA 12.0 respectively based on the random-effect model if statistical heterogeneity was great [12], otherwise, the fixed–effect model was adopted [13]. In addition, we employed sensitivity analysis, Begg’s funnel plot and Egger’s test [14] to determine the impact of individual study on the overall result and potential publication bias respectively. All *P* values were two-sided and below 0.05 was regarded as statistical significance.

## Results

### Search results and description of studies

**Fig 1:**
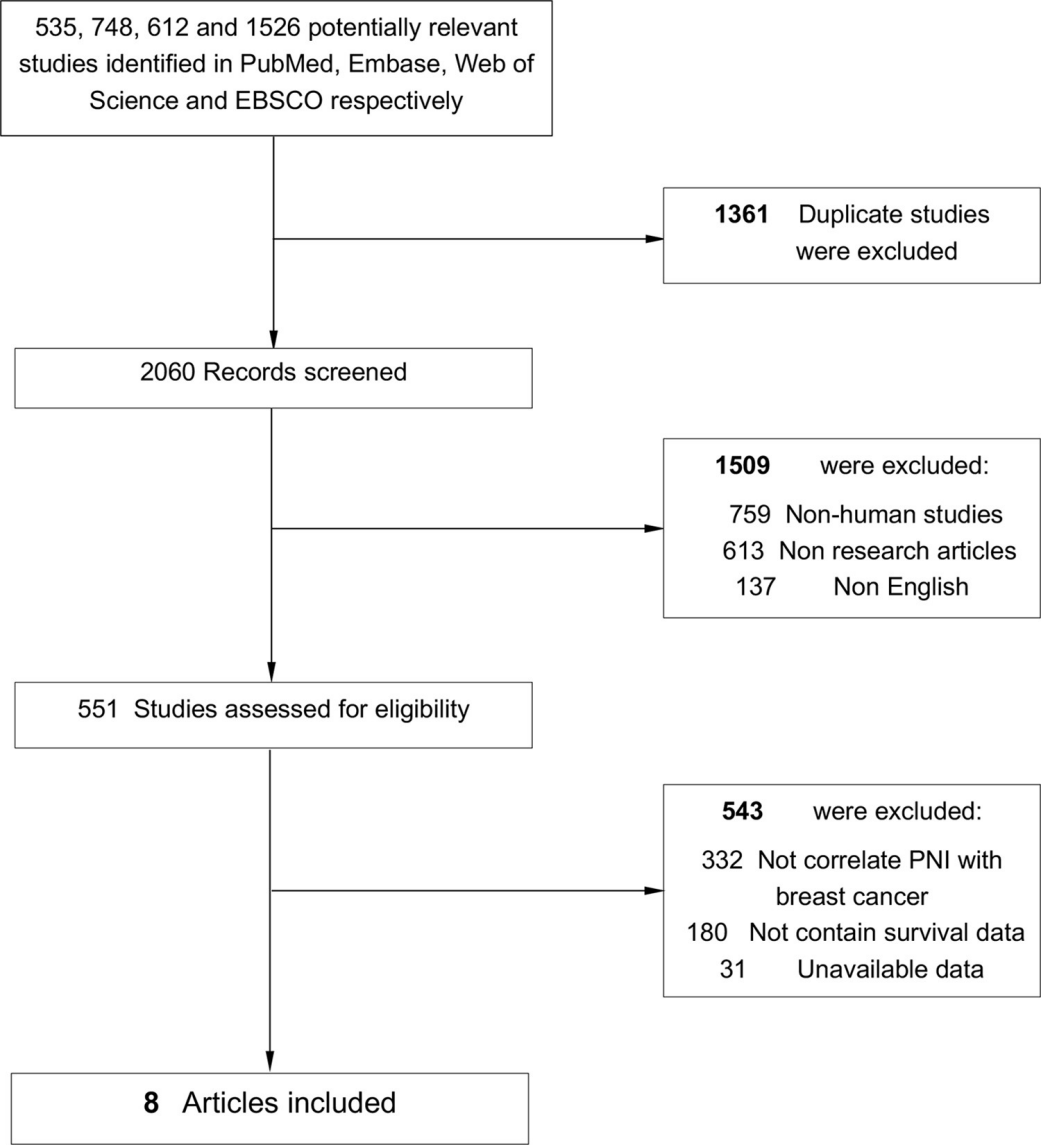
Flow chart diagram of study selection.

Flow chart diagram of study selection was shown in Fig 1. Eight studies with 2322 patients were finally included in this meta-analysis [3, 8–10, 15–18]. And all these studies were scored 6 or above after careful evaluation with the Newcastle–Ottawa Scale (NOS). (S1 and S2 Tables in S1 File) Four studies evaluated both OS and DFS outcomes; and two studies evaluated only OS, whereas two studies evaluated only DFS. PNI was calculated from pretreatment laboratory data in all these included studies. Characteristics of researches being appropriate for data integration were exhibited in Table 1 and S3 Table in S1 File.

**Table 1:** 

Study	Year	Patients’ No.	M / F	median age (range) (year)	Method for evaluation of PNI	Cut-off value	PNI: (H/L)	TNM stage	median follow-up (months)	Clinical outcome	Quality Score (NOS)
Oba, T. et al [15]	2021	60	0/60	NR	10 × serum albumin (g/dl) + 0.005 × total lymphocyte count (per mm3)	NR	27/33	IV	11.6	OS	7
Soberanis-Piña, P.D. et al [16]	2021	110	0/110	NR	10 × serum albumin (g/dl) + 0.005 × total lymphocyte count (per mm3)	≥ 32.1	48/62	Ⅰ- Ⅲ	65	5-Year DFS	7
Chen, L. et al [10]	2021	785	0/785	55 (25–83)	serum albumin (ALB) (g/L) + 5 × total lymphocyte count (109/L)	≥ 51	532/253	Ⅰ- Ⅲ	NR	OS, DFS	8
Oba, T. et al [8]	2020	191	0/191	1.2 ± 10.4	10 × serum albumin (g/dl) + 0.005 × total lymphocyte count (per mm3)	NR	82/109	Ⅰ- Ⅲ	51 (1, 151)	OS, DFS	8
Wang, Y.H. et al [3]	2019	202	0/202	≥57: 76.9%; <57: 23.1%	serum albumin (ALB) (g/L) + total lymphocyte count (109/L)	≥ 45	55/147	Ⅱ-Ⅲ	26 (16, 42)	DFS	7
Hua, X. et al [9]	2019	380	0/380	47 (26, 78)	10 × serum albumin (g/dl) + 0.005 × total lymphocyte count (per mm3)	≥ 52	247/133	Ⅱ	63.1 (3.2, 95.9)	OS	8
Mohri, T. et al [17]	2016	212	0/212	66 (27, 96)	10 × serum albumin (g/dl) + 0.005 × total lymphocyte count (per mm3)	≥ 52.8	85/127	Ⅰ- Ⅲ	47.7	OS, DFS	7
Yang, Z.J. et al [18]	2014	382	0/382	50 (22, 85)	10 × serum albumin (g/dl) + 0.005 × total lymphocyte count (per mm3)	≥ 48.7	243/139	Ⅱ-Ⅲ	74 (6, 101)	OS, DFS	8

PNI, prognostic nutritional index; OS, overall survival; DFS, disease–free survival; H, high; L, low; NR, not reported.

### Meta-analyses

#### Overall survival (OS)

**Fig 2:**
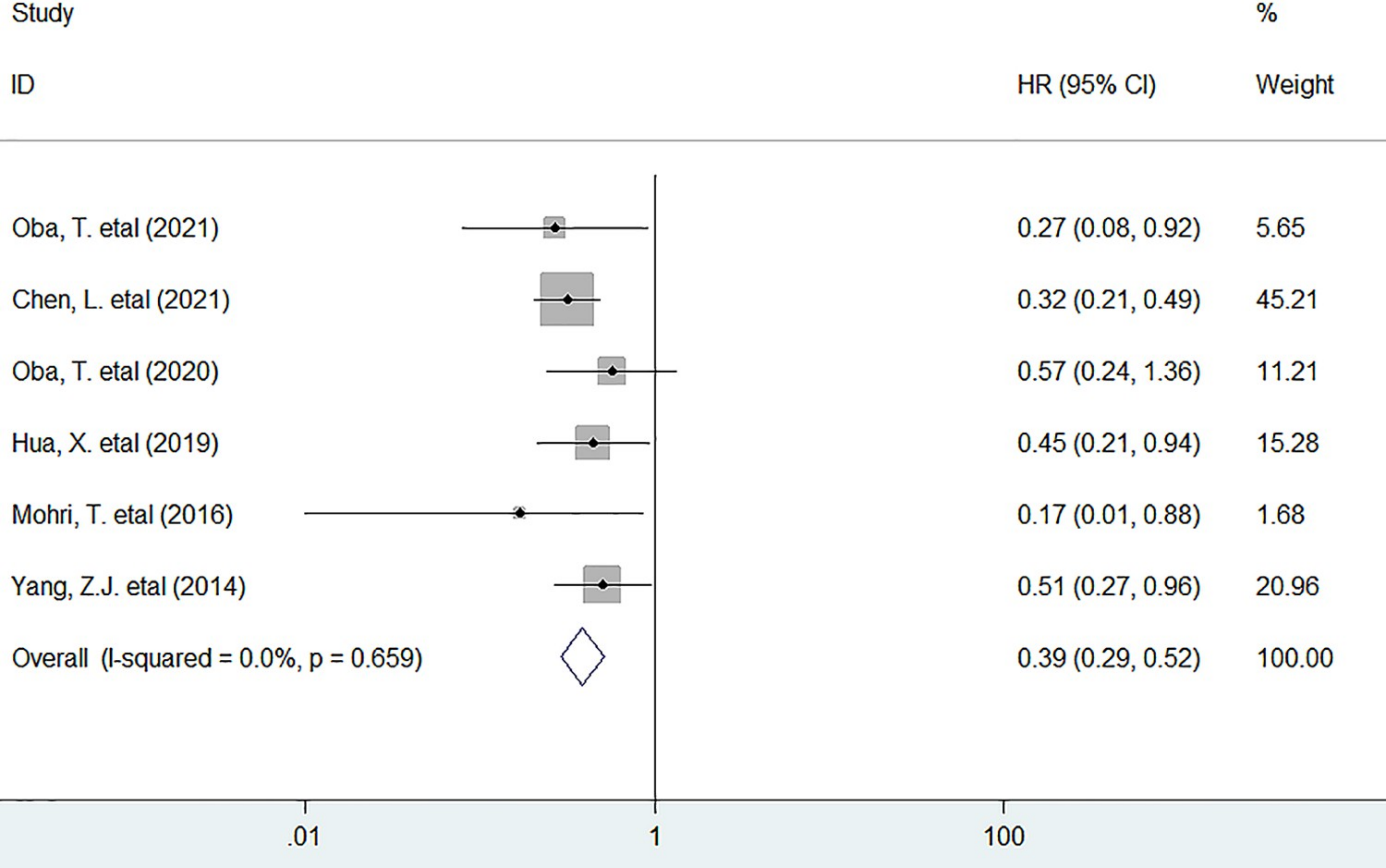
Forest plots describing HR of the association between low baseline PNI and OS in breast cancer patients. HR: hazard ratio; PNI, prognostic nutritional index; OS: overall survival.

Six studies involving 2010 patients investigated the association between PNI and OS, and the meta-analysis indicated that low PNI before treatment remarkably decreased OS (HR = 0.39, 95% CI 0.29 to 0.52, *P <* 0.001) in patients with BC, with no heterogeneity being detected (*I*^*2*^ *= 0*.*0%*, *P = 0*.*659*) (Fig 2).

**Fig 3:**
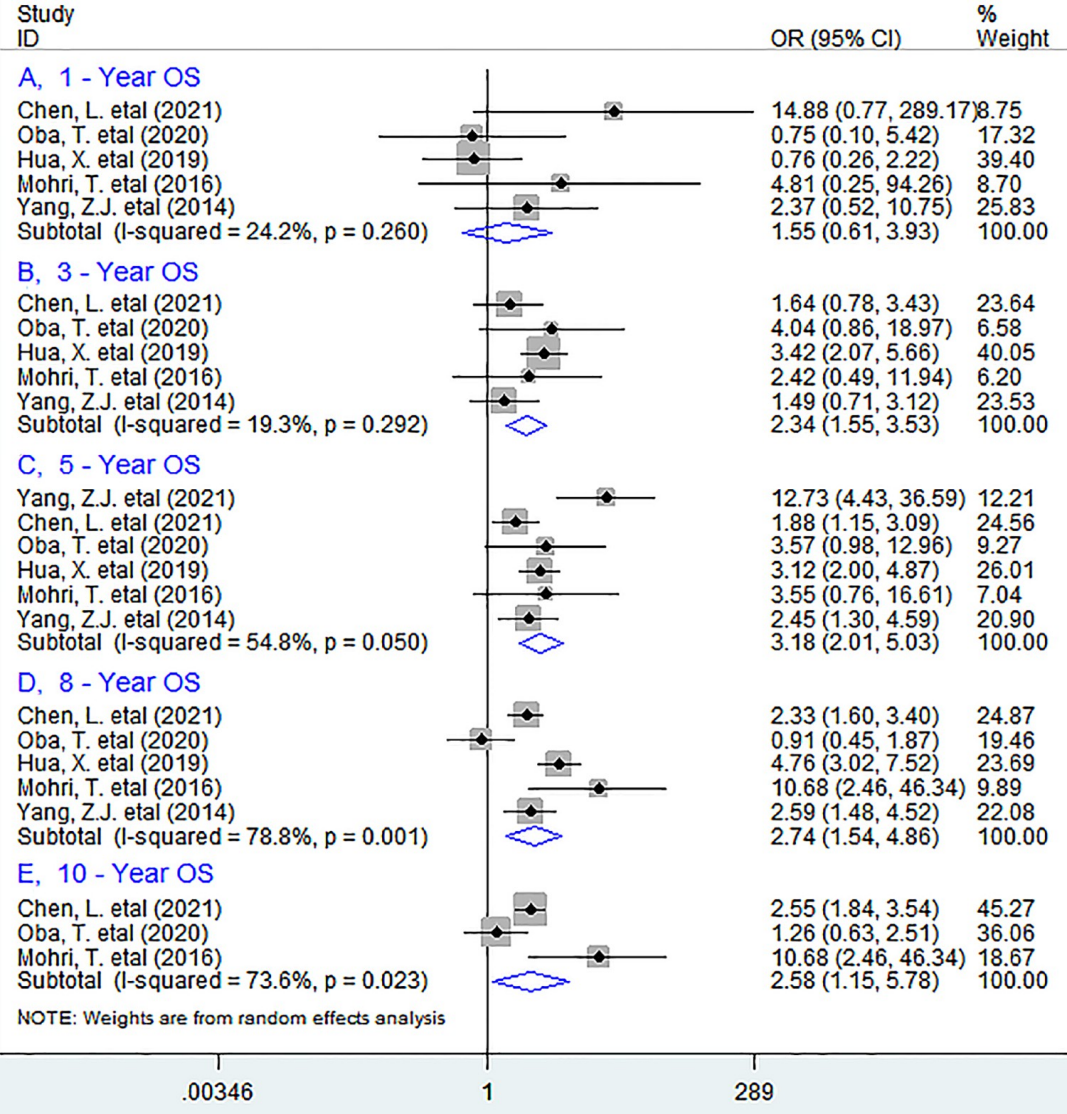
Forest plots describing ORs of the association between low baseline PNI and OS at 1-year(A), 3-year(B), 5-year(C), 8-year(D) and 10-year(E) in breast cancer patients. OR, odds ratios; PNI, prognostic nutritional index; OS, overall survival.

In addition, in subgroup analyses according to the specific survival time, we noted that low pretreatment PNI was significantly associated with worse 3-year (OR = 2.34, 95% CI 1.55 to 3.53, *P <* 0.001), 5-year (OR = 3.18, 95% CI 2.01 to 5.03, *P <* 0.001), 8-year (OR = 2.74, 95% CI 1.54 to 4.86, *P* = 0.001) and 10-year (OR = 2.58, 95% CI 1.15 to 5.78, *P* = 0.021) OS, but not with 1-year survival rate (OR = 1.55, 95% CI 0.61 to 3.93, *P* = 0.353) (Fig 3).

#### Disease–free survival (DFS)

**Fig 4:**
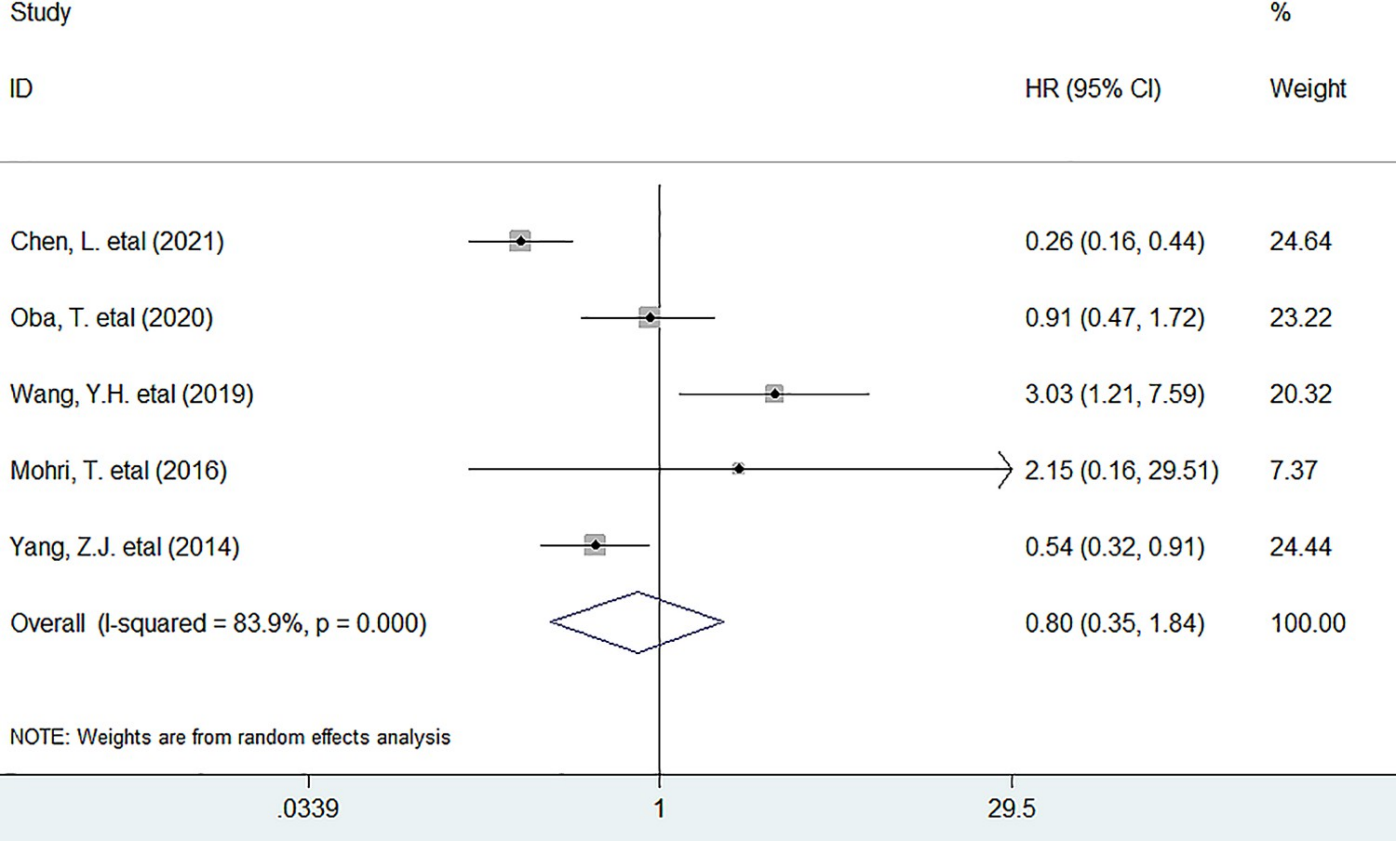
Forest plots describing HRs of the association between decreased baseline PNI and DFS in breast cancer patients. HR: hazard ratio; PNI, prognostic nutritional index; DFS, disease–free survival.

As for the association between PNI and DFS, five studies have supplied the relevant data. In this study, we discovered that reduced pretreatment PNI could not markedly deteriorate DFS in BC patients (HR = 0.80, 95% CI 0.35 to 1.84, *P* = 0.605) (Fig 4).

**Fig 5:**
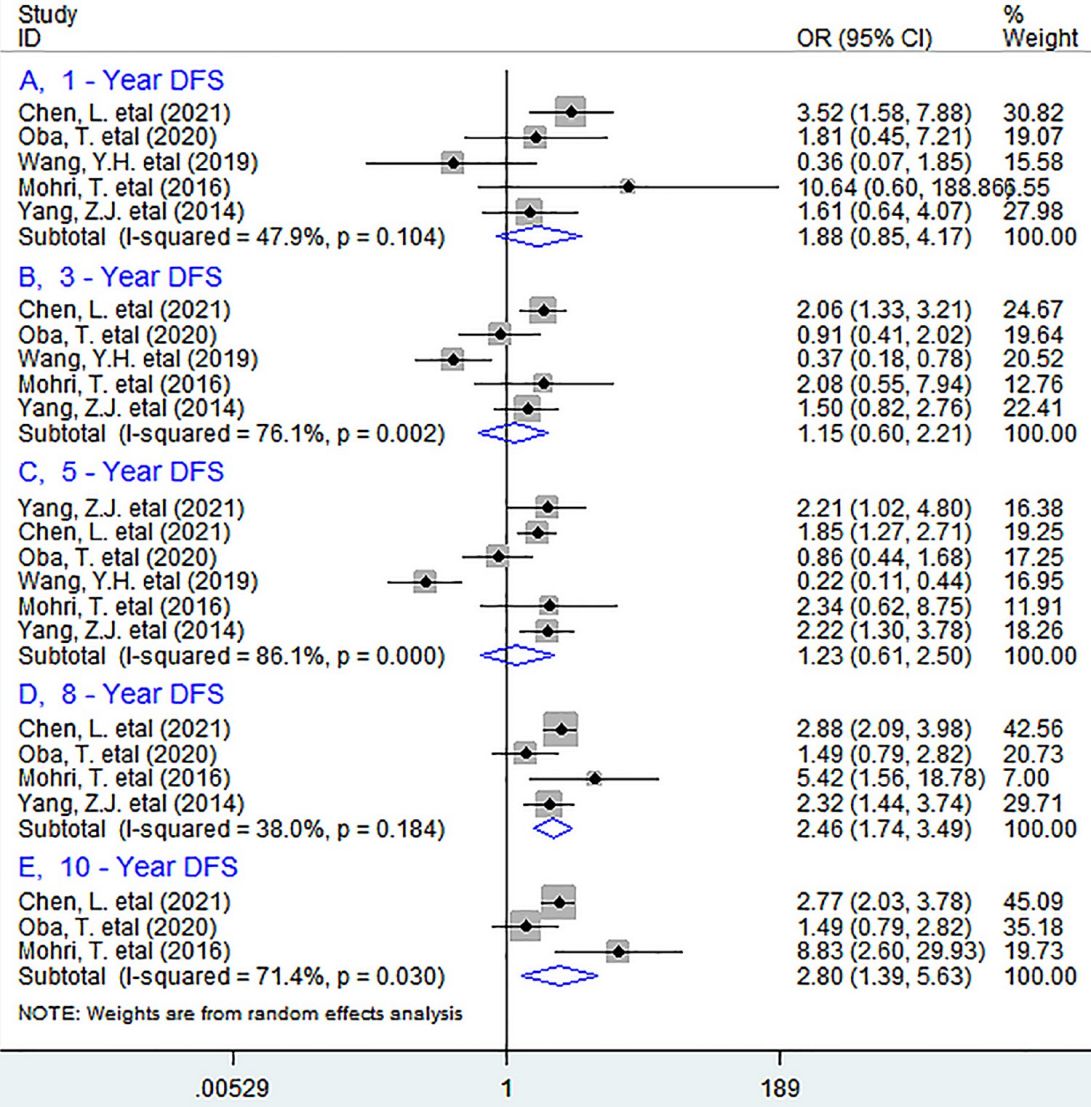
Forest plots describing ORs of the association between reduced baseline PNI and DFS at 1-year (A), 3-year(B), 5-year(C), 8-year(D) and 10-year(E) in breast cancer patients. OR, odds ratios; PNI, prognostic nutritional index; DFS, disease–free survival.

In subgroup analyses, as shown in Fig 5, poor PNI before treatment considerably decreased 8-year (OR = 2.46, 95% CI 1.74 to 3.49, *P <* 0.001) and 10-year (OR = 2.80, 95% CI 1.39 to 5.63, *P* = 0.004) DFS rather than 1-year (OR = 1.88, 95% CI 0.85 to 4.17, *P* = 0.120), 3-year (OR = 1.15, 95% CI 0.60 to 2.21, *P* = 0.664), or 5-year (OR = 1.23, 95% CI 0.61 to 2.50, *P =* 0.560) DFS in BC.

#### Clinicopathological features

We next investigated whether PNI before treatment correlated with clinicopathological features, however, the results indicated that it was not significantly associated with ER (OR = 0.89, 95% CI 0.70 to 1.14, *P* = 0.355), PR (OR = 0.88, 95% CI 0.69 to 1.11, *P* = 0.279), or Her-2 (OR = 1.13, 95% CI 0.79 to 1.62, *P* = 0.501) status of patients (S1 Fig in S1 File).

#### Sensitivity analysis

Sensitivity analysis demonstrated that each included research had no impact on the overall result for OS or DFS (S2 Fig in S1 File).

#### Publication bias

Funnel plot and Egger’s test indicated that no significant publication bias existed between PNI and OS (*P* = 0.926) or DFS (*P* = 0.261) in patients (S3 Fig in S1 File).

## Discussion

Nutritional immune status is closely related to antitumor response and survival of cancer patients [19]. PNI, an indicator which can reflect the nutritional immune status of patients, has proven to be associated with prognosis in multiple of solid tumors. However, the role of PNI in human BC still remains inconclusive. In this study, we unraveled the relationship between elevated pretreatment PNI and survival of human BC, and elaborated that it was significantly associated with better 3-year, 5-year, 8-year and 10-year OS, but not with 1-year survival rate in BC. In addition, it remarkably ameliorated 8-year and 10-year DFS, rather than 1-year, 3-year or 5-year DFS in patients. These findings suggested that high baseline PNI played an important role in hindering tumor progression and metastasis of BC.

Although the association between reduced PNI and worse survival of BC was probably complex and largely unknown, several potential mechanisms might be responsible for this. PNI was derived from the absolute albumin and absolute lymphocyte counts and combined both nutrition and systemic inflammation status. A low PNI suggests a reduction in serum albumin and /or lymphocytes, serving as surrogate biomarker. Serum albumin was an important nutritional indicator and Lower level of serum albumin was indicative of systemic responses that resulted in the loss of proteins [20, 21]. Serum albumin has been adopted to appraise disease severity, progression and been considered as an independent factor of poor prognosis in varieties of cancers including breast carcinoma [22].

Lymphocyte count from circulating blood has proven to be an important index in preventing tumor progression of cancers via activating host immune response. For example, CD8^+^T cells exerted an important role in immune surveillance and anti-tumor response through secreting multiples of cytokines such as perforin, granzyme B [23]. While CD4^+^T-helper cells could promote anti-tumor immunity via the release of cytokines including IFN-γ [24]. In addition, the reduction in level of lymphocytes in peripheral blood would lead to the decrease of these cells in infiltration of tumors, and weakened the response to cytotoxic treatment thereby lowering the survival of cancer [25–27]. Taken together, a decrease in albumin or lymphocyte count in the peripheral blood links to tumor initiation and progression. Thus, low PNI might confer advantages of survival and invasion by tumor cells and result in worse outcome.

The strengths of this study were that it included the application of systematic literature review as a methodology and eliminated the risk for miscounting study subjects; in addition, it also included increased power and improved estimates of effect size; and more importantly, it reached much reliable conclusions among included individual studies with conflicting results. However, there were some limitations in this meta-analysis. First, the cut-off value used for definition of high PNI in included Individual studies are not inconsistent. Second, significant heterogeneity observed across studies in pooled analysis of DFS can’t be completely accounted despite the use of random-effect models. Finally, studies with negative results might not be published, which could give rise to potential publication bias.

In conclusion, poor pretreatment PNI deteriorated OS, 8-year and 10-year DFS in BC patients, implicating that it is a valuable prognostic index and improving the nutritional immune status may offer a therapeutic strategy for these patients.

## Supporting information

S1 Checklist PRISMA 2009 checklist. (DOC)

S1 File (DOC)

## Abbreviations

PNI: prognostic nutritional index
BC: breast cancer
OS: overall survival
DFS: disease–free survival
ER: estrogen receptor
PR: progesterone receptor
HR: hazard rations
OR: odds ratios
Cl: confidence interval
NR: not reported
