# Supplementary material for: Low pretreatment prognostic nutritional index predicts poor survival in breast cancer patients: A meta-analysis
Source: PLoS One. 2023 Jan 20;18(1):e0280669. doi: 10.1371/journal.pone.0280669 (PMC9858712; doi:10.1371/journal.pone.0280669)
Supplement: S1 File — (DOC) [file pone.0280669.s002.doc]

**Supplementary data**

**Low Pretreatment Prognostic Nutritional Index Predicts Poor Survival In Breast Cancer Patients: A Meta-analysis**

Guoming Hu ^1, 2,3^ *, Qiannan Ding ^4^, Kefang Zhong ^1^, Shimin Wang ^5^, Songxiang Wang^1^, Liming Huang ^1^, *

^1^ Department of General Surgery (Breast and Thyroid Surgery), Shaoxing People’s Hospital; Shaoxing Hospital, Zhejiang University School of Medicine; Shaoxing, Zhejiang, China.

^2^ Key Laboratory of Cancer Prevention and Intervention, Ministry of Education, Hangzhou, Zhejiang, China.

^3^ Shaoxing Key Laboratory of Functional Molecular Imaging of Tumor and Interventional Diagnosis and Treatment, Shaoxing, Zhejiang, China.

^4^ Medical Research Center, Shaoxing People’s Hospital; Shaoxing Hospital, Zhejiang University School of Medicine; Shaoxing, Zhejiang, China.

^5^ Department of Nephrology, Shaoxing People’s Hospital; Shaoxing Hospital, Zhejiang University School of Medicine; Shaoxing, Zhejiang, China.

***Corresponding author:**

**E-mail:** Guoming Hu: hgmplj@126.com (GMH);

Songxiang Wang: wangsongxiang8@163.com (SXW);

Liming Huang: shaoxinghlm@126.com (LMH).


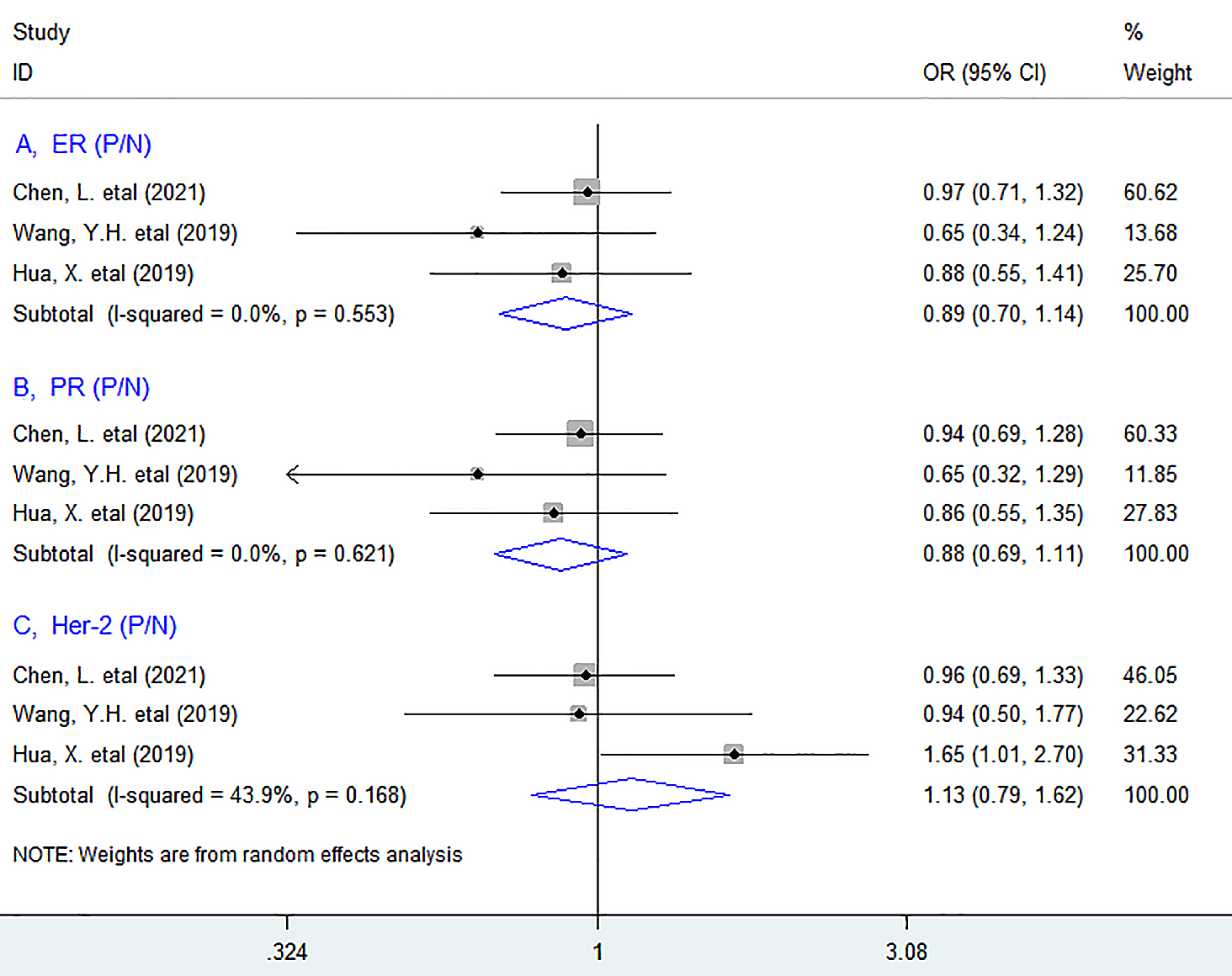


**Fig. S1.** Forest plots indicating ORs of the association between low baseline PNI and clinicopathological features including ER (**A**), PR (**B**) and Her-2 status (**C**) in patients. OR, odds ratios; PNI, prognostic nutritional index; ER, Estrogen Receptor; PR, Progesterone receptor; P: positive; N: negative.

**
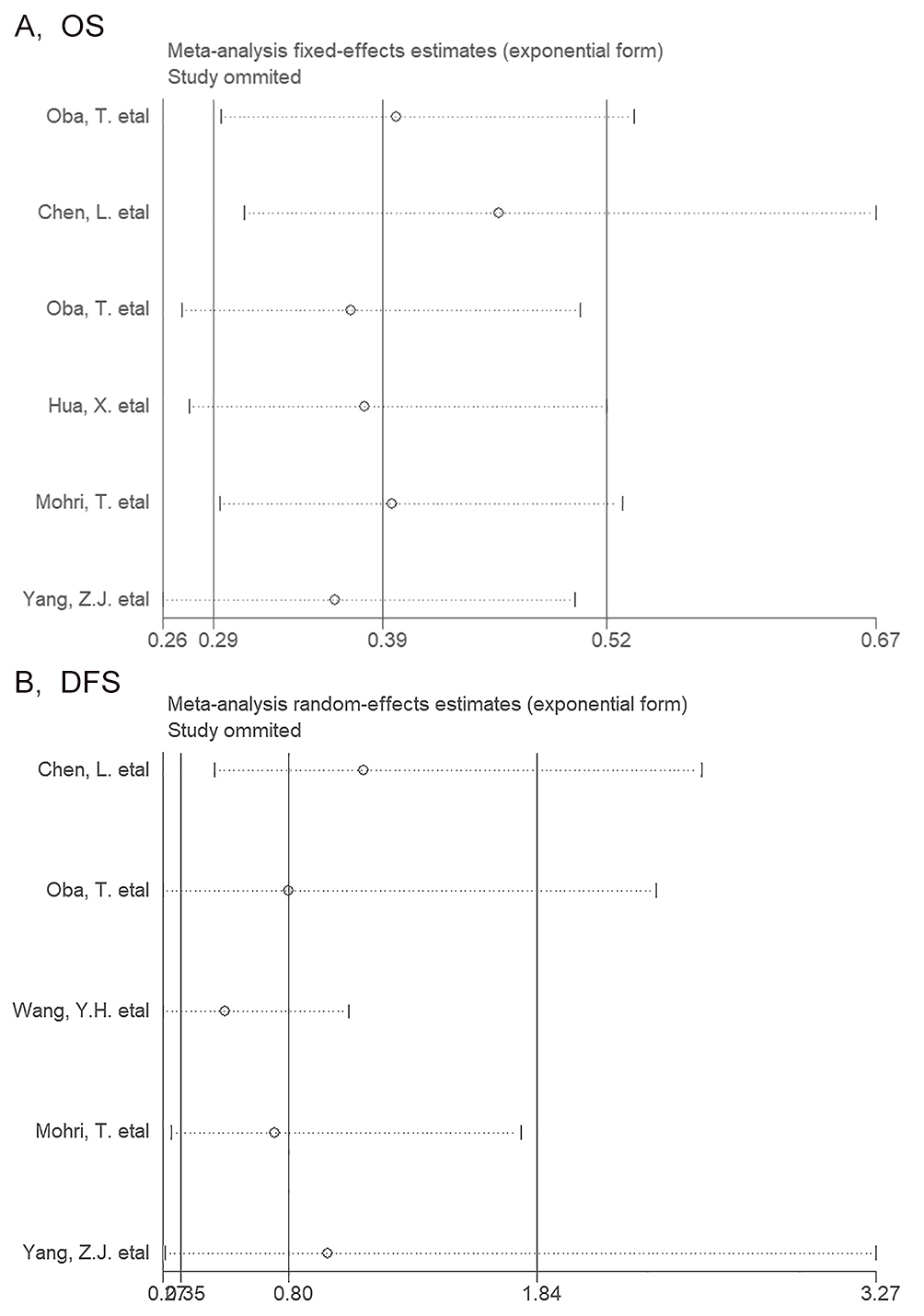
**

**Fig. S2.** Plots describing the influence of individual studies on the overall HRs for OS (**A**) and DFS (**B**) in patients. OS: overall survival; DFS, disease**–**free survival.

**
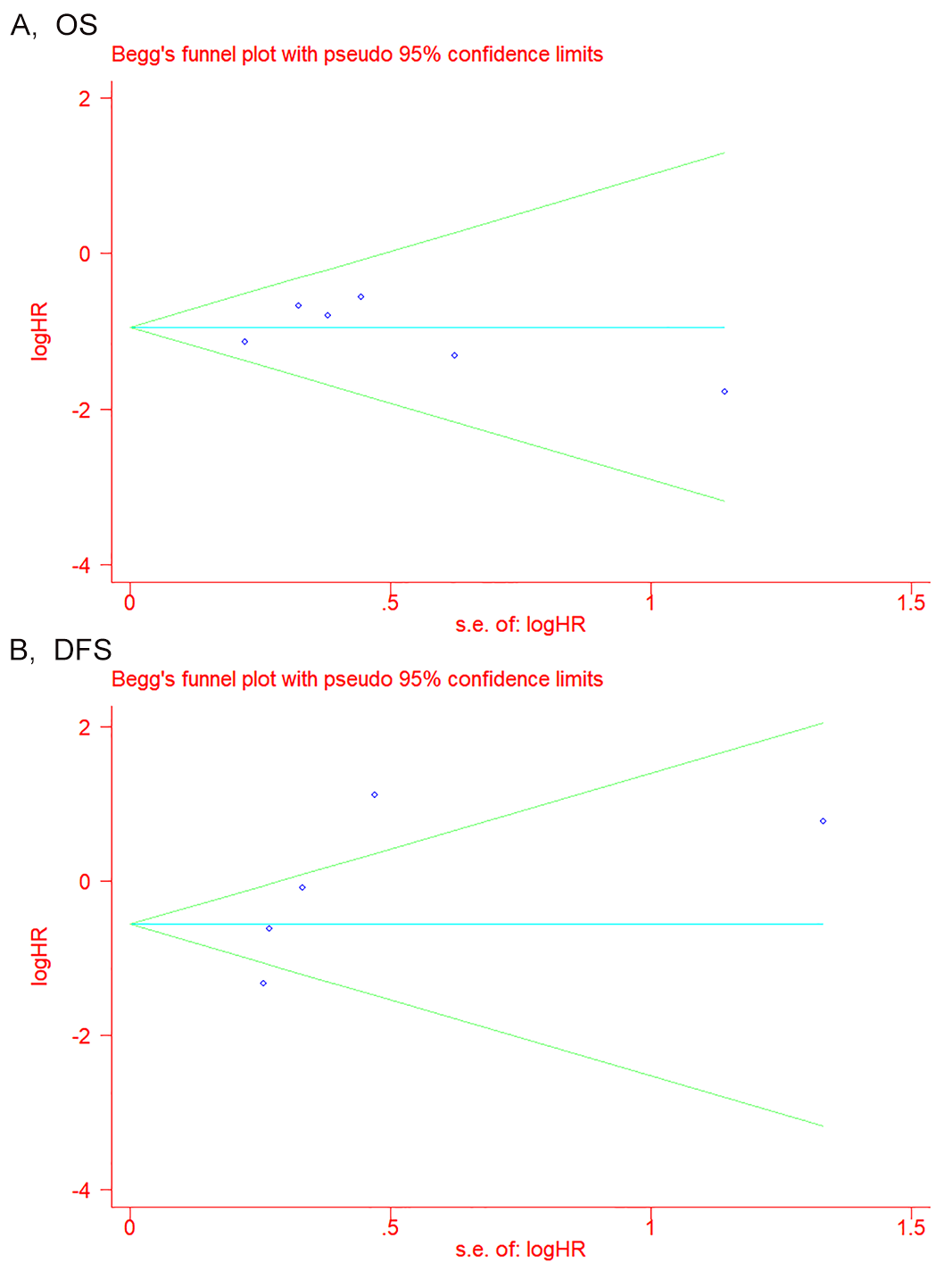
**

**Fig. S3.** Funnel plots displayed the potential publication bias between decreased baseline PNI and OS (**A**) or DFS (**B**) in patients. PNI, prognostic nutritional index; OS: overall survival; DFS, disease**–**free survival.

**New Table S1.** Specific criteria of Newcastle-Ottawa Scale (NOS)

| **NOTE**: Identify ‘high’ quality choices with a ‘star’ (one ‘star’ means 1 score ); A maximum of one ‘star’ for each item within the *‘Selection’* and *‘Outcome’* categories; maximum of two ‘stars’ for *‘Comparability’.* Using the tool, each study is judged on eight items, categorized into three groups: the ‘selection’ of the study groups; the ‘comparability’ of the groups; and the ascertainment of the outcome of interest for cohort studies. Stars awarded for each quality item serve as a quick visual assessment. Stars are awarded such that the highest quality studies are awarded up to nine stars. |
| --- |
| **1, Selection** (4) |
| ⑴，Representativeness of the exposed cohort |
| a) truly representative of the average ____ (describe) in the community ★  b) somewhat representative of the average ____ in the community ★  c) selected group of users eg nurses, volunteers  d) no description of the derivation of the cohort |
| ⑵，Selection of the non exposed cohort |
| a) drawn from the same community as the exposed cohort ★  b) drawn from a different source  c) no description of the derivation of the non exposed cohort |
| ⑶，Ascertainment of exposure to implants |
| a) secure record (eg surgical records) ★  b) structured interview ★  c) written self report  d) no description |
| ⑷，Demonstration that outcome of interest was not present at start of study |
| a) yes ★  b) no |
| **2, Comparability** (1) |
| ⑴， Comparability of cohorts on the basis of the design or analysis |
| a) study controls for ______ (select the most important factor) ★  b) study controls for any additional factor (This criteria could be modified to indicate specific control for a second important factor.) ★ |
| **3, Outcome** (3) |
| ⑴， Assessment of outcome |
| a) independent blind assessment ★  b) record linkage ★  c) self report  d) no description |
| ⑵， Was follow up long enough for outcomes to occur |
| a) yes (select an adequate follow up period for outcome of interest) ★  b) no |
| ⑶， Adequacy of follow up of cohorts |
| a) complete follow up - all subjects accounted for ★  b) subjects lost to follow up unlikely to introduce bias - small number lost - > ___ % (select an adequate %) follow up, or description of those lost) ★  c) follow up rate < ___% (select an adequate %) and no description of those lost  d) no statement |

**New Table S2.** Study quality assessment according to the Newcastle–Ottawa Scale (NOS)

| **Study** | **Year** | **Selection** | | | | **Comparability** | | **Outcome** | | | **Quality Score (NOS)** |
| --- | --- | --- | --- | --- | --- | --- | --- | --- | --- | --- | --- |
|  |  | **⑴** | **⑵** | ⑶ | ⑷ | **⑴ - a** | **⑴ - b** | **⑴** | **⑵** | ⑶ |  |
| Oba, T. etal [15] | 2021 | Yes | Yes | Yes | Yes | Yes | No | Yes | No | Yes | 7 |
| Soberanis-Piña, P.D. etal [16] | 2021 | Yes | Yes | Yes | No | No | Yes | Yes | Yes | Yes | 7 |
| Chen, L. etal [10] | 2021 | Yes | Yes | Yes | Yes | Yes | No | Yes | Yes | Yes | 8 |
| Oba, T. etal [8] | 2020 | Yes | Yes | Yes | Yes | No | Yes | Yes | Yes | Yes | 8 |
| Wang, Y.H. etal [3] | 2019 | Yes | Yes | Yes | No | Yes | Yes | Yes | No | Yes | 7 |
| Hua, X. etal [9] | 2019 | Yes | Yes | Yes | Yes | No | Yes | Yes | Yes | Yes | 8 |
| Mohri, T. etal [17] | 2016 | Yes | Yes | Yes | Yes | No | No | Yes | Yes | Yes | 7 |
| Yang, Z.J. etal [18] | 2014 | Yes | Yes | Yes | Yes | Yes | No | Yes | Yes | Yes | 8 |

**Table S3.** Characteristics of the included studies for OR analysis of clinicopathological features.

| **Research** | **Year** | **No. of Patients** | **PNI: (H/L)** | **Tumor stage** | **ER (P / N)** | **PR (P / N)** | **Her-2 (P / N)** |
| --- | --- | --- | --- | --- | --- | --- | --- |
| Chen, L. etal [10] | 2021 | 784 | 532/253 | Ⅰ- Ⅲ | H:(330/202) L:(159/94) | H:(316/216) L:(154/99) | H:(153/379) L:(75/178) |
| Wang, Y.H. etal [3] | 2019 | 202 | 55/147 | Ⅱ-Ⅲ | H:(34/21) L:(105/42) | H:(38/17) L:(114/33) | H:(22/33) L:(61/86) |
| Hua, X. etal [9] | 2019 | 380 | 247/133 | Ⅱ | H:(173/71) L:(97/35) | H:(158/86) L:(90/42) | H:(79/145) L:(31/94) |

PNI: prognostic nutritional index; H: high; L: low; P: positive; N: negative; ER, estrogen receptor; PR, progesterone receptor.
